# Using quantitative and qualitative data in health services research – what happens when mixed method findings conflict? [ISRCTN61522618]

**DOI:** 10.1186/1472-6963-6-28

**Published:** 2006-03-08

**Authors:** Suzanne Moffatt, Martin White, Joan Mackintosh, Denise Howel

**Affiliations:** 1 Public Health Research Group, School of Population & Health Sciences, Faculty of Medical Sciences, William Leech Building, Framlington Place, Newcastle upon Tyne NE2 4HH, UK

## Abstract

**Background:**

In this methodological paper we document the interpretation of a mixed methods study and outline an approach to dealing with apparent discrepancies between qualitative and quantitative research data in a pilot study evaluating whether welfare rights advice has an impact on health and social outcomes among a population aged 60 and over.

**Methods:**

Quantitative and qualitative data were collected contemporaneously. Quantitative data were collected from 126 men and women aged over 60 within a randomised controlled trial. Participants received a full welfare benefits assessment which successfully identified additional financial and non-financial resources for 60% of them. A range of demographic, health and social outcome measures were assessed at baseline, 6, 12 and 24 month follow up. Qualitative data were collected from a sub-sample of 25 participants purposively selected to take part in individual interviews to examine the perceived impact of welfare rights advice.

**Results:**

Separate analysis of the quantitative and qualitative data revealed discrepant findings. The quantitative data showed little evidence of significant differences of a size that would be of practical or clinical interest, suggesting that the intervention had no impact on these outcome measures. The qualitative data suggested wide-ranging impacts, indicating that the intervention had a positive effect. Six ways of further exploring these data were considered: (i) treating the methods as fundamentally different; (ii) exploring the methodological rigour of each component; (iii) exploring dataset comparability; (iv) collecting further data and making further comparisons; (v) exploring the process of the intervention; and (vi) exploring whether the outcomes of the two components match.

**Conclusion:**

The study demonstrates how using mixed methods can lead to different and sometimes conflicting accounts and, using this six step approach, how such discrepancies can be harnessed to interrogate each dataset more fully. Not only does this enhance the robustness of the study, it may lead to different conclusions from those that would have been drawn through relying on one method alone and demonstrates the value of collecting both types of data within a single study. More widespread use of mixed methods in trials of complex interventions is likely to enhance the overall quality of the evidence base.

## Background

Combining quantitative and qualitative methods in a single study is not uncommon in social research, although, 'traditionally a gulf is seen to exist between qualitative and quantitative research with each belonging to distinctively different paradigms'. [1] Within health research there has, more recently, been an upsurge of interest in the combined use of qualitative and quantitative methods, sometimes termed mixed methods research [2] although the terminology can vary. [3] Greater interest in qualitative research has come about for a number of reasons: the numerous contributions made by qualitative research to the study of health and illness [4-6]; increased methodological rigor [7] within the qualitative paradigm, which has made it more acceptable to researchers or practitioners trained within a predominantly quantitative paradigm [8]; and, because combining quantitative and qualitative methods may generate deeper insights than either method alone. [9] It is now widely recognised that public health problems are embedded within a range of social, political and economic contexts. [10] Consequently, a range of epidemiological and social science methods are employed to research these complex issues. [11] Further legitimacy for the use of qualitative methods alongside quantitative has resulted from the recognition that qualitative methods can make an important contribution to randomised controlled trials (RCTs) evaluating complex health service interventions. There is published work on the various ways that qualitative methods are being used in RCTs (e.g. [12,13] but little on how they can optimally enhance the usefulness and policy relevance of trial findings. [14,15]

A number of mixed methods publications outline the various ways in which qualitative and quantitative methods can be combined. [1,2,9,16] For the purposes of this paper with its focus on mixed methods in the context of a pilot RCT, the significant aspects of mixed methods appear to be: purpose, process and, analysis and interpretation. In terms of purpose, qualitative research may be used to help identify the relevant variables for study [17], develop an instrument for quantitative research [18], to examine different questions (such as acceptability of the intervention, rather than its outcome) [19]; and to examine the same question with different methods (using, for example participant observation or in depth interviews [1]). Process includes the priority accorded to each method and ordering of both methods which may be concurrent, sequential or iterative. [20] Bryman [9] points out that, 'most researchers rely primarily on a method associated with either quantitative or qualitative methods and then buttress their findings with a method associated with the other tradition' (p128). Both datasets may be brought together at the 'analysis/interpretation' phase, often known as 'triangulation' [21]. Brannen [1] suggests that most researchers have taken this to mean more than one type of data, but she stresses that Denzin's original conceptualisation involved methods, data, investigators or theories. Bringing different methods together almost inevitably raises discrepancies in findings and their interpretation. However, the investigation of such differences may be as illuminating as their points of similarity. [1,9]

Although mixed methods are now widespread in health research, quantitative and qualitative methods and results are often published separately. [22,23] It is relatively rare to see an account of the methodological implications of the strategy and the way in which both methods are combined when interpreting the data within a particular study. [1] A notable exception is a study showing divergence between qualitative and quantitative findings of cancer patients' quality of life using a detailed case study approach to the data. [13]

By presenting quantitative and qualitative data collected within a pilot RCT together, this paper has three main aims: firstly, to demonstrate how divergent quantitative and qualitative data led us to interrogate each dataset more fully and assisted in the interpretation process, producing a greater research yield from each dataset; secondly, to demonstrate how combining both types of data at the analysis stage produces 'more than the sum of its parts'; and thirdly, to emphasise the complementary nature of qualitative and quantitative methods in RCTs of complex interventions. In doing so, we demonstrate how the combination of quantitative and qualitative data led us to conclusions different from those that would have been drawn through relying on one or other method alone.

The study that forms the basis of this paper, a pilot RCT to examine the impact of welfare rights advice in primary care, was funded under the UK Department of Health's Policy Research Programme on tackling health inequalities, and focused on older people. To date, little research has been able to demonstrate how health inequalities can be tackled by interventions within and outside the health sector. Although living standards have risen among older people, a common experience of growing old is worsening material circumstances. [24] In 2000–01 there were 2.3 million UK pensioners living in households with below 60 per cent of median household income, after housing costs. [25] Older people in the UK may be eligible for a number of income- or disability-related benefits (the latter could be non-financial such as parking permits or adaptations to the home), but it has been estimated that approximately one in four (about one million) UK pensioner households do not claim the support to which they are entitled. [26] Action to facilitate access to and uptake of welfare benefits has taken place outside the UK health sector for many years and, more recently, has been introduced within parts of the health service, but its potential to benefit health has not been rigorously evaluated. [27-29]

## Methods

There are a number of models of mixed methods research. [2,16,30] We adopted a model which relies of the principle of complementarity, using the strengths of one method to enhance the other. [30] We explicitly recognised that each method was appropriate for different research questions. We undertook a pragmatic RCT which aimed to evaluate the health effects of welfare rights advice in primary care among people aged over 60. Quantitative data included standardised outcome measures of health and well-being, health related behaviour, psycho-social interaction and socio-economic status ; qualitative data used semi-structured interviews to explore participants' views about the intervention, its outcome, and the acceptability of the research process.

Following an earlier qualitative pilot study to inform the selection of appropriate outcome measures [31], contemporaneous quantitative and qualitative data were collected. Both datasets were analysed separately and neither compared until both analyses were complete. The sampling strategy mirrored the embedded design; probability sampling for the quantitative study and theoretical sampling for the qualitative study, done on the basis of factors identified in the quantitative study.

Approval for the study was obtained from Newcastle and North Tyneside Joint Local Research Ethics Committee and from Newcastle Primary Care Trust.

### The intervention

The intervention was delivered by a welfare rights officer from Newcastle City Council Welfare Rights Service in participants' own homes and comprised a structured assessment of current welfare status and benefits entitlement, together with active assistance in making claims where appropriate over the following six months, together with necessary follow-up for unresolved claims.

### Quantitative study

The design presented ethical dilemmas as it was felt problematic to deprive the control group of welfare rights advice, since there is adequate evidence to show that it leads to significant financial gains. [32] To circumvent this dilemma, we delivered welfare rights advice to the control group six months after the intervention group. A single-blinded RCT with allocation of individuals to intervention (receipt of welfare rights consultation immediately) and control condition (welfare rights consultation six months after entry into the trial) was undertaken.

Four general practices located at five surgeries across Newcastle upon Tyne took part. Three of the practices were located in the top ten per cent of most deprived wards in England using the Index of Multiple Deprivation (two in the top one percent – ranked 30^th ^and 36^th ^most deprived); the other practice was ranked 3,774 out of a total of 8,414 in England. [33]

Using practice databases, a random sample of 100 patients aged 60 years or over from each of four participating practices was invited to take part in the study. Only one individual per household was allowed to participate in the trial, but if a partner or other adult household member was also eligible for benefits, they also received welfare rights advice. Patients were excluded if they were permanently hospitalised or living in residential or nursing care homes.

Written informed consent was obtained at the baseline interview. Structured face to face interviews were carried out at baseline, six, 12 and 24 months using standard scales covering the areas of demographics, mental and physical health (SF36) [34], Hospital Anxiety and Depression Scale (HADS) [35], psychosocial descriptors (e.g. Social Support Questionnaire [36] and the Self-Esteem Inventory, [37], and socioeconomic indicators (e.g. affordability and financial vulnerability). [38] Additionally, a short semi-structured interview was undertaken at 24 months to ascertain the perceived impact of additional resources for those who received them.

All health and welfare assessment data were entered onto customised MS Access databases and checked for quality and completeness. Data were transferred to the Statistical Package for the Social Sciences (SPSS) v11.0 [39] and STATA v8.0 for analysis. [40]

### Qualitative study

The qualitative findings presented in this paper focus on the impact of the intervention. The sampling frame was formed by those (n = 96) who gave their consent to be contacted during their baseline interview for the RCT. The study sample comprised respondents from intervention and control groups purposively selected to include those eligible for the following resources: financial only; non-financial only; both financial and non financial; and, none. Sampling continued until no new themes emerged from the interviews; until data 'saturation' was reached. [21]

Initial interviews took place between April and December 2003 in participants' homes after their welfare rights assessment; follow-up interviews were undertaken in January and February 2005. The semi-structured interview schedule covered perceptions of: impact of material and/or financial benefits; impact on mental and/or physical health; impact on health related behaviours; social benefits; and views about the link between material resources and health. All participants agreed to the interview being audio-recorded. Immediately afterwards, observational field notes were made. Interviews were transcribed in full.

Data analysis largely followed the framework approach. [41] Data were coded, indexed and charted systematically; and resulting typologies discussed with other members of the research team, 'a pragmatic version of double coding'. [42] Constant comparison [43] and deviant case analysis [44] were used since both methods are important for internal validation. [7,42] Finally, sets of categories at a higher level of abstraction were developed.

A brief semi-structured interview was undertaken (by JM) with all participants who received additional resources. These interview data explored the impact data of additional resources on all of those who received them, not just the qualitative sub-sample. The data were independently coded by JM and SM using the same coding frame. Discrepant codes were examined by both researchers and a final code agreed.

## Results

### Quantitative study

One hundred and twenty six people were recruited into the study; there were 117 at 12 month follow-up and 109 at 24 months (five deaths, one moved, the remainder declined).

Table 1 shows the distribution of financial and non-financial benefits awarded as a result of the welfare assessments. Sixty percent of participants were awarded some form of welfare benefit, and just over 40% received a financial benefit. Some households received more than one type of benefit.

Table 2 compares the quantitative and qualitative sub-samples on a number of personal, economic, health and lifestyle factors at baseline. Intervention and control groups were comparable.

Table 3 compares outcome measures by award group, i.e. no award, non-financial and financial and shows only small differences between the mean changes across each group, none of which were statistically significant. Other analyses of the quantitative data compared the changes seen between baseline and six months (by which time the intervention group had received the welfare rights advice but the control group had not) and found little evidence of differences between the intervention and control groups of any practical importance. The only statistically significant difference between the groups was a small decrease in financial vulnerability in the intervention group after six months. [45]

There was little evidence for differences in health and social outcomes measures as a result of the receipt of welfare advice of a size that would be of major practical or clinical interest. However, this was a pilot study, with only the power to detect large differences if they were present. One reason for a lack of difference may be that the scales were less appropriate for older people and did not capture all relevant outcomes. Another reason for the lack of differences may be that insufficient numbers of people had received their benefits for long enough to allow any health outcomes to have changed when comparisons were made. Fourteen per cent of participants found to be eligible for financial benefits had not started receiving their benefits by the time of the first follow-up interview after their benefit assessment (six months for intervention, 12 months for control); and those who had, had only received them for an average of 2 months. This is likely to have diluted any impact of the intervention effect, and might account, to some extent, for the lack of observed effect.

### Qualitative study

Twenty five interviews were completed, fourteen of whom were from the intervention group. Ten participants were interviewed with partners who made active contributions. Twenty two follow-up interviews were undertaken between twelve and eighteen months later (three individuals were too ill to take part).

Table 1 (fifth column) shows that 14 of the participants in the qualitative study received some financial award. The median income gain was (€84, $101) (range £10 (€15, $18) -£100 (€148, $178)) representing a 4%-55% increase in weekly income. 18 participants were in receipt of benefit, either as a result of the current intervention or because of claims made prior to this study.

By the follow-up (FU) interviews all but one participant had been receiving their benefits for between 17 and 31 months. The intervention was viewed positively by all interviewees irrespective of outcome. However, for the fourteen participants who received additional financial resources the impact was considerable and accounts revealed a wide range of uses for the extra money. Participants' accounts revealed four linked categories, summarised on Table 4. Firstly, increased affordability of *necessities*, without which maintaining independence and participating in daily life was difficult. This included accessing transport, maintaining social networks and social activities, buying better quality food, stocking up on food, paying bills, preventing debt and affording paid help for household activities. Secondly, *occasional expenses *such as clothes, household equipment, furniture and holidays were more affordable. Thirdly, extra income was used to act as a cushion against potential *emergencies *and to increase *savings*. Fourthly, all participants described the easing of financial worries as bringing '*peace of mind'*.

Without exception, participants were of the view that extra money or resources would not improve existing health problems. The reasons behind these strongly held views about individual health conditions was generally that their poor health was attributed to specific health conditions and a combination of family history or fate, which were immune to the effects of money. Most participants had more than one chronic condition and felt that because of these conditions, plus their age, additional money would have no effect.

However, a number of participants linked the impact of the intervention with improved ways of coping with their conditions because of what the extra resources enabled them to do:

Mrs T: *Having money is not going to improve his health, we could win the lottery and he would still have his health problems. *

Mr T: *No, but we don't need to worry if I wanted *.... *Well I mean I eat a lot of honey and I think it's very good, very healthful for you *... *at one time we couldn't have afforded to buy these things. Now we can go and buy them if I fancy something, just go and get it where we couldn't before*. 

Mrs T: *Although the Attendance Allowance is actually his [partners], it's made me relax a bit more *...*I definitely worry less now *(N15, female, 62 and partner)

Despite the fact that no-one expected their own health conditions to improve, most people believed that there was a link between resources and health in a more abstract sense, either because they experienced problems affording necessities such as healthy food or maintaining adequate heat in their homes, or because they empathised with those who lacked money. Participants linked adequate resources to maintaining health and contributing to a sense of well-being.

*Money does have a lot to do with health if you are poor. It would have a lot to do with your health *... *I don't buy loads and loads of luxuries, but I know I can go out and get the food we need and that sort of thing. I think that money is a big part of how a house, or how people in that house are*. (N13, female, 72)

### Comparing the results from the two datasets

When the separate analyses of the quantitative and qualitative datasets after the 12 month follow-up structured interviews were completed, the discrepancy in the findings became apparent. The quantitative study showed little evidence of a size that would be of practical or clinical interest, suggesting that the intervention had no impact on these outcome measures. The qualitative study found a wide-ranging impact, indicating that the intervention had a positive effect. The presence of such inter-method discrepancy led to a great deal of discussion and debate, as a result of which we devised six ways of further exploring these data.

#### (i) Treating the methods as fundamentally different

This process of simultaneous qualitative and quantitative dataset interrogation enables a deeper level of analysis and interpretation than would be possible with one or other alone and demonstrates how mixed methods research produces more than the sum of its parts. It is worth emphasising however, that it is not wholly surprising that each method comes up with divergent findings since each asked different, but related questions, and both are based on fundamentally different theoretical paradigms. Brannen [1] and Bryman [9] argue that it is essential to take account of these theoretical differences and caution against taking a purely technical approach to the use of mixed methods, a simple 'bolting together' of techniques. [17] Combining the two methods for crossvalidation (triangulation) purposes is not a viable option because it rests on the premise that both methods are examining the same research problem. [1] We have approached the divergent findings as indicative of different aspects of the phenomena in question and searched for reasons which might explain these inconsistencies. In the approach that follows, we have treated the datasets as complementary, rather than attempt to integrate them, since each approach reflects a different view on how social reality ought to be studied.

#### (ii) Exploring the methodological rigour of each component

It is standard practice at the data analysis and interpretation phases of any study to scrutinise methodological rigour. However, in this case, we had another dataset to use as a yardstick for comparison and it became clear that our interrogation of each dataset was informed to some extent by the findings of the other. It was not the case that we expected to obtain the same results, but clearly the divergence of our findings was of great interest and made us more circumspect about each dataset. We began by examining possible reasons why there might be problems with each dataset individually, but found ourselves continually referring to the results of the other study as a benchmark for comparison.

With regard to the quantitative study, it was a pilot, of modest sample size, and thus not powered to detect small differences in the key outcome measures. In addition there were three important sources of dilution effects: firstly, only 63% of intervention group participants received some type of financial award; secondly, we found that 14% of those in the trial eligible for financial benefits did not receive their money until after the follow up assessments had been carried out; and thirdly, many had received their benefits for only a short period, reducing the possibility of detecting any measurable effects at the time of follow-up. All of these factors provide some explanation for the lack of a *measurable *effect between intervention and control group and between those who did and did not receive additional financial resources.

The number of participants in the qualitative study who received additional financial resources as a result of this intervention was small (n = 14). We would argue that the fieldwork, analysis and interpretation [46] were sufficiently transparent to warrant the degree of methodological rigour advocated by Barbour [7,17] and that the findings were therefore an accurate reflection of what was being studied. However, there still remained the possibility that a reason for the discrepant findings was due to differences between the qualitative sub-sample and the parent sample, which led us to step three.

#### (iii) Exploring dataset comparability

We compared the qualitative and quantitative samples on a number of social and economic factors (Table 2). In comparison to the parent sample, the qualitative sub-sample was slightly older, had fewer men, a higher proportion with long-term limiting illness, but fewer current smokers. However, there was nothing to indicate that such small differences would account for the discrepancies. There were negligible differences in SF-36 (Physical and Mental) and HAD (Anxiety and Depression) scores between the groups at baseline, which led us to discount the possibility that those in the quantitative sub sample were markedly different to the quantitative sample on these outcome measures.

#### (iv) Collection of additional data and making further comparisons

**Figure 1:**
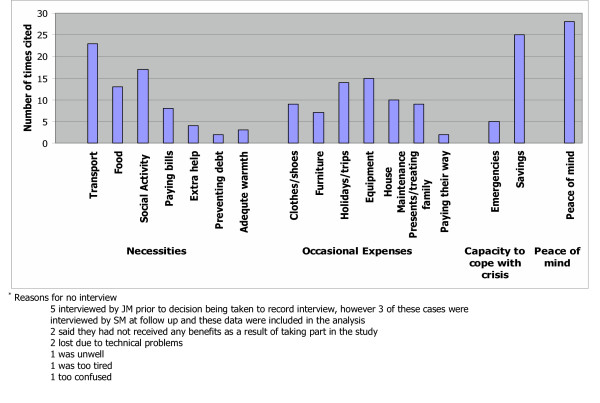
Use of additional resources at 2 year follow up (N = 35)*.

The divergent findings led us to seek further funding to undertake collection of additional quantitative and qualitative data at 24 months. The quantitative and qualitative follow-up data verified the initial findings of each study. [45] We also collected a limited amount of qualitative data on the perceived impact of resources, from all participants who had received additional resources. These data are presented in figure 1 which shows the uses of additional resources at 24 month follow-up for 35 participants (N = 35, 21 previously in quantitative study only, 14 in both). This dataset demonstrates that similar issues emerged for both qualitative and quantitative datasets: transport, savings and 'peace of mind' emerged as key issues, but the data also showed that the additional money was used on a wide range of items. This follow-up confirmed the initial findings of each study and further, indicated that the perceived impact of the additional resources was the same for a larger sample than the original qualitative sub-sample, further confirming our view that the positive findings extended beyond the fourteen participants in the qualitative sub-sample, to all those receiving additional resources.

#### (v) Exploring whether the intervention under study worked as expected

The qualitative study revealed that many participants had received welfare benefits via other services prior to this study, revealing the lack of a 'clean slate' with regard to the receipt of benefits, which we had not anticipated. We investigated this further in the quantitative dataset and found that 75 people (59.5%) had received benefits prior to the study; if the first benefit was on health grounds, a later one may have been because their health had deteriorated further.

#### (vi) Exploring whether the outcomes of the quantitative and qualitative components match

'Probing certain issues in greater depth' as advocated by Bryman (p134) [1] focussed our attention on the outcome measures used in the quantitative part of the study and revealed several challenges. Firstly, the qualitative study revealed a number of dimensions not measured by the quantitative study, such as, 'maintaining independence' which included affording paid help, increasing and improving access to facilities and managing better within the home. Secondly, some of the measures used with the intention of capturing dimensions of mental health did not adequately encapsulate participants' accounts of feeling 'less stressed' and 'less depressed' by financial worries. Probing both datasets also revealed congruence along the dimension of physical health. No differences were found on the SF36 physical scale and participants themselves did not expect an improvement in physical health (for reasons of age and chronic health problems). The real issue would appear to be measuring ways in which older people are better able to cope with existing health problems and maintain their independence and quality of life, despite these conditions.

Qualitative study results also led us to look more carefully at the quantitative measures we used. Some of the standardised measures were not wholly applicable to a population of older people. Mallinson [47] also found this with the SF36 when she demonstrated some of its limitations with this age group, as well as how easy it is to, 'fall into the trap of using questionnaires like a form of laboratory equipment and forget that ... they are open to interpretation'. The data presented here demonstrate the difficulties of trying to capture complex phenomena quantitatively. However, they also demonstrate the usefulness of having alternative data forms on which to draw whether complementary (where they differ but together generate insights) or contradictory (where the findings conflict). [30] In this study, the complementary and contradictory findings of the two datasets proved useful in making recommendations for the design of a definitive study.

## Discussion

Many researchers understand the importance, indeed the necessity, of combining methods to investigate complex health and social issues. Although quantitative research remains the dominant paradigm in health services research, qualitative research has greater prominence than before and is no longer, as Barbour [42] points out regarded as the 'poor relation to quantitative research that it has been in the past' (p1019). Brannen [48] argues that, despite epistemological differences there are 'more overlaps than differences'. Despite this, there is continued debate about the authority of each individual mode of research which is not surprising since these different styles, 'take institutional forms, in relation to cultures of and markets for knowledge' (p168). [49] Devers [50] points out that the dominance of positivism, especially within the RCT method, has had an overriding influence on the criteria used to assess research which has had the inevitable result of viewing qualitative studies unfavourably. We advocate treating qualitative and quantitative datasets as complementary rather than in competition for identifying the true version of events. This, we argue, leads to a position which exploits the strengths of each method and at the same time counters the limitations of each. The process of interpreting the meaning of these divergent findings has led us to conclude that much can be learned from scientific realism [51]which has 'sought to position itself as a model of scientific explanation which avoids the traditional epistemological poles of positivism and relativism' (p64). This stance enables investigators to take account of the complexity inherent in social interventions and reinforces, at a theoretical level, the problems of attempting to measure the impact of a social intervention via experimental means. However, the current focus on evidence based health care [52] now includes public health [53,54] and there is increased attention paid to the results of trials of public health interventions, attempting as they do, to capture complex social phenomena using standardised measurement tools. We would argue that at the very least, the inclusion of both qualitative and quantitative elements in such studies, is essential and ultimately more cost-effective, increasing the likelihood of arriving at a more thoroughly researched and better understood set of results.

## Conclusion

The findings of this study demonstrate how the use of mixed methods can lead to different and sometimes conflicting accounts. This, we argue, is largely due to the outcome measures in the RCT not matching the outcomes emerging from the qualitative arm of the study. Instead of making assumptions about the *correct *version, we have reported the results of both datasets together rather than separately, and advocate six steps to interrogate each dataset more fully. The methodological strategy advocated by this approach involves contemporaneous qualitative and quantitative data collection, analysis and reciprocal interrogation to inform interpretation in trials of complex interventions. This approach also indicates the need for a realistic appraisal of quantitative tools. More widespread use of mixed methods in trials of complex interventions is likely to enhance the overall quality of the evidence base.

## Competing interests

The author(s) declare that they have no competing interests.

## Authors' contributions

SM and MW had the original idea for the study, and with the help of DH, Adam Sandell and Nick Whitton developed the proposal and gained funding. JM collected the data for the quantitative study, SM designed and collected data for the qualitative study. JM, DH and MW analysed the quantitative data, SM analysed the qualitative data. All authors contributed to interpretation of both datasets. SM wrote the first draft of the paper, JM, MW and DH commented on subsequent drafts. All authors have read and approved the final manuscript.

## Pre-publication history

The pre-publication history for this paper can be accessed here:



## Figures and Tables

**Table 1:** Distribution of financial and non-financial benefit awards made to study participants by group allocation

**Type of Award**	**Number (%) of intervention** ** group (n = 59)**	**Number (%) of control** ** group (n = 58)**	**Number (%) of participants ** ** (n = 117)**	**Qualitative study ** **sub-sample ** **(n = 25)**
Received no award	22 (37.3)	25 (43.1)	47 (40.2)	7*
Received some type of award(s)	37 (62.7)	33 (56.9)	70 (59.8)	18
Received only financial award(s)	13 (22.0)	19 (32.8)	32 (27.4)	7
Received only non-financial award(s)	12 (20.3)	7 (12.1)	19 (16.2)	4
Received both financial and non-financial awards	12 (20.3)	7 (12.1)	19 (16.2)	7

* At first interview, it was found that four participants received an award prior to the study

**Table 2:** Comparison between participants in quantitative and qualitative components

	**Quantitative Group** ** (n = 126)**	**Qualitative Study Sub-Sample ** **(n = 25)**
Mean age (range)	70.6	74.6
% Males	56.3	44.0
% Living as a couple	55.6	52.0
% In Council Tax Bands A & B*	72.2	76.0
% Long term limiting illness	64.3	76.0
% Current smokers	19.1	8.0
SF-36 Physical Mean Score	27.45	25.22
SF-36 Mental	51.44	53.55
HAD-Anxiety	5.86	5.88
HAD-Depression	4.49	3.24

* Living in residence of low value

**Table 3:** Comparison of changes at 6 months after welfare rights assessment* by award group

	**No benefits ** **(n = 46)**	**Non-financial benefit ** **(n=21)**	**Financial benefit ** **(n = 50)**	
		
	**Mean (SD)**	**Mean (SD)**	**Mean (SD)**	P Value**
**Health**				
SF36 – Physical	0.8 (7.6)	-0.1 (5.5)	1.9 (6.6)	0.5
SF36 – Mental	0.8 (8.3)	1.7 (7.5)	0.3 (7.0)	0.8
HAD – Anxiety	-0.4 (1.9)	-0.6 (2.7)	-0.1 (2.8)	0.7
HAD – Depression	0.3 (2.5)	1.6 (2.3)	1.1 (2.5)	0.1
**Health related behaviour**				
Fruit and vegetable intake	0.5 (8.3)	-0.6 (7.6)	0.1 (9.9)	0.9
Protein intake	0.7 (5.1)	-0.5 (5.8)	1.2 (5.0)	0.5
Alcohol (units)	-1.1 (12.6)	-1.4 (9.4)	0.1 (12.4)	0.9
**Psycho-social**				
Social interaction	-0.2 (3.2)	-0.1 (3.6)	0.2 (3.0)	0.8
Self Esteem Inventory	0.5 (4.1)	-0.1 (3.0)	0.4 (3.3)	0.3
**Socio-economic**				
Financial vulnerability	-0.7 (3.0)	-0.3 (3.0)	-1.3 (2.7)	0.3
Standard of Living Index	0.4 (0.8)	0.1 (0.8)	0.1 (1.0)	0.4

*Changes are over 0–6 months in intervention group and 6–12 months in control group

**Testing for equal mean changes using ANOVA

**Table 4:** Summary of findings from qualitative study

**Necessities**
*I go down to town on the bus. I've got a four wheel trolley, it takes a lot of shopping ... I get a taxi home every week and it really does help. I don't have to think about it*.(N14, female, 84, FU) *I eat a lot more fruit and better food really*. (N3, female, 74, FU)
**One-off payments**
*My fridge-freezer was on the blink, it's useless having these things mended, I didn't know when I would get one, but when I looked and saw I'd got the money back to April, I could go out and get one straight away and pay cash for it, it was an absolutely wonderful feeling*. (N14, female, 84) *I had a four day holiday in Wales ... it's the first holiday I've had in thirty years ... it was wonderful, where before you know I would never, never been able to afford it*. (N3, female 74, FU)
**Emergencies**
*We haven't got that much money left in the bank now ... so, yes, it does help for emergencies*. (N15, female, 62)
**Peace of mind**
*I used to get so depressed that I couldn't afford this that and the other, and now you know, I think to myself, oooh look at my statement I can afford that, that's lovely, it makes me feel good*. (N14, female, 82)
